# Long-Term Association Between Patient-Reported Outcomes and Psychological Factors in Patients With a Distal Radius Fracture

**DOI:** 10.1016/j.jhsg.2024.06.004

**Published:** 2024-07-02

**Authors:** Viktor Schmidt, Cecilia Tervaniemi, Mats Wadsten

**Affiliations:** ∗ Department of Diagnostics and Intervention, Umeå University, Umeå, Sweden

**Keywords:** Distal radius fracture, Long-term results, Outcomes, PROM, Psychological factors

## Abstract

**Purpose:**

The outcome after a distal radius fracture (DRF) is often evaluated with radiography, clinical examination, and patient-reported outcome measures. However, research has identified associations between psychological factors and outcomes after a DRF. A knowledge gap exists about psychological factors and their potential implications for long-term outcomes after a DRF. The aim of this study was to examine the long-term association between psychological factors and patient-reported outcomes.

**Methods:**

This multicenter investigation included patients aged 15–75 years with closed physes presenting with an acute DRF. Patients who completed a long-term follow-up (after 11–13 years) with patient-reported outcome measures were invited to participate in the study, and surveys measuring psychological factors were sent to the patients.

**Results:**

Two hundred and four patients (70%) completed the follow-up (mean [range] age at injury, 56 [18–75] years; 154 were females [75%]). Multivariable analysis showed that higher age, injury to the dominant hand, and greater pain catastrophizing were associated with an increase in scores on the Disabilities of the Arm, Shoulder, and Hand questionnaire.

**Conclusions:**

A decade after sustaining a DRF, patients with higher scores on the Pain Catastrophizing Scale reported inferior outcomes as measured by the Disabilities of the Arm, Shoulder, and Hand. The Pain Catastrophizing Scale accounts for 13% of the observed variance in Disabilities of the Arm, Shoulder, and Hand.

**Type of study/level of evidence:**

Therapeutic level IIb.

Distal radius fractures (DRFs) are the most common fracture type.^1^^,^^2^ Outcomes after DRFs are often evaluated with radiographs, range of motion, grip strength, and patient-reported outcome measures (PROMs).^3^ Although a few studies have examined long-term outcomes after a DRF, most have focused on the correlation among PROMs, radiographs, and treatment.^4^^,^^5^ Studies on short-term outcomes after a DRF have reported a correlation between malunion and function.^6^^,^^7^ Other factors associated with the outcome after a DRF are income, injury compensation, education, and physiotherapy.^8^, ^9^, ^10^ Although not routinely evaluated, research has established a correlation between psychosocial factors and PROMs following a DRF, as well as other musculoskeletal conditions such as chronic shoulder pain.^11^^,^^12^ The Pain Catastrophizing Scale (PCS), General Self-Efficacy Scale (GSE) and Hospital Anxiety and Depression Scale (HADS) are widely used questionnaires to assess the association between psychological factors and PROMs.^11^^,^^13^, ^14^, ^15^, ^16^, ^17^, ^18^, ^19^, ^20^ Studies have found an association between the GSE and function after DRF surgery and total knee replacement.^13^^,^^14^

Moreover, depression is linked to complications after orthopaedic surgery.^15^, ^16^, ^17^, ^18^ Finally, several studies have found that PROMs after a DRF are associated with PCS scores.^11^^,^^19^^,^^20^ Most studies on psychological factors are conducted on short-term outcomes after injury. A knowledge gap exists about psychological factors and their potential implications for long-term outcomes after a DRF. Therefore, this study examines the long-term association between psychological factors and clinical outcomes.

## Materials and Methods

### Study design and setting

Between October 2009 and September 2011, patients with a DRF were invited to participate in a prospective study conducted at two hospitals (Sundsvall and Östersund) in Sweden.^7^^,^^21^ Previous studies have examined the relationship between clinical outcomes and their short- and long-term association with radiographic outcomes.^5^^,^^7^ The current study examines the long-term results of psychological factors and their association with PROMs. Sundsvall and Östersund hospitals are classified as secondary level hospitals providing medical services to a combined catchment area of 300,000 residents. The study was conducted in accordance with the STROBE (STrengthening the Reporting of OBservational studies in Epidemiology) guidelines.

### Participants and data collection

Of 451 patients initially included in the cohort, there were 324 eligible patients at a 10-year follow-up. Of these 324 patients, 292 (90%) completed a long-term follow-up (11–13 years) with PROMs. The participants in this study were invited to examine psychological factors and PROMs following a DRF (Fig.).

**Figure:**
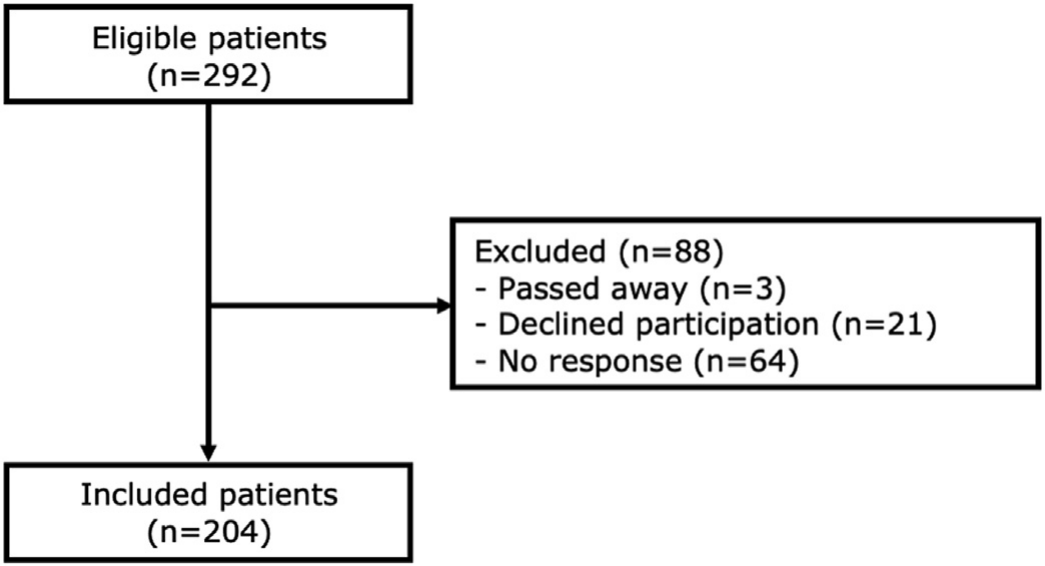
Flowchart of patients included in the study.

An envelope containing information, a consent form, three surveys, and a prepaid return envelope were sent to all 292 eligible patients. Missing items were collected by telephone when possible. In other cases, missing items were substituted using person-mean imputation (person-mean substitution, PMS).^22^^,^^23^

### Outcome measures

Quick Disabilities of the Arm, Shoulder, and Hand (*Quick*DASH) is a shorter version of the Disabilities of the Arm, Shoulder, and Hand questionnaire.^24^ *Quick*DASH contains 11 items on function and symptoms of the upper extremity, with a total score ranging from 0 points, indicating no disability, to 100 points, indicating most disability.

Psychological factors were analyzed using the self-reported GSE, the PCS, and the HADS.^25^, ^26^, ^27^ The GSE measures a person’s perceived ability to manage future situations and obstacles. The scale comprises 10 items, each of which is rated by patients on a 4-point Likert scale from 1 (not likely at all) to 4 (exactly true), resulting in a total score ranging from 10 to 40 points. A higher score indicates greater self-efficacy.^25^ The PCS, which comprises 13 items and three subscales, measures pain. Each item is rated on a 5-point Likert scale from 0 (never) to 4 (all the time). The subscales gauge the extent of helplessness (inability to deal with painful situations), rumination (worry), and magnification (expectancies for negative outcomes). Higher scores are associated with higher pain catastrophizing.^26^ The HADS measures anxiety and depression on two subscales (HADS-A and HADS-D, representing anxiety and depression), each containing seven items on a 4-point Likert scale from 0 to 3 with a total score ranging from 0 to 21 points for each subscale: < 7 points signify a lower likelihood for depression/anxiety, 8–10 points the possibility of depression/anxiety and > 11 points a higher probability of depression/anxiety.^27^

### Implants and surgery

All patients were treated according to an algorithm.^7^ Both surgically and nonsurgically treated patients were included.

### Ethical approval

Ethical approval for this study was obtained from the regional ethical committee at Umeå University (Dnr 09-213 and 2021-03224).

### Statistics

Descriptive statistics included frequencies and percentages, mean and range, medians and interquartile ranges. The Wilcoxon rank-sum test and Spearman rank correlation were employed for bivariate analysis. Data were assessed for multicollinearity, with all correlation matrices < 0.60 and the variance inflation factor < 1.1. In constructing the linear regression model, variables were examined based on their significance in bivariate analysis, with a threshold of *P* < .10. Variables were included if they increased the adjusted R^2^ of the model.

All analyses were performed with R (v 4.3.2) and knitr (v. 1.45) for reproducible research as well as sensemaker (v. 0.1.4), arsenal (v. 3.6.3) and ggplot2 (v. 3.4.4) for tables and plots.

## Results

### Patients and descriptive data

Of 292 eligible patients, 204 (70%) were included in the study (flow chart, Fig.). Of these 204 patients, 154 (75%) were female. The age at injury ranged from 18 to 75 years (with a mean of 50 years for men and 58 years for women) (Table 1).

**Table 1:** Patient Demographics for Responders and Nonresponders

Variable	Responders (n = 204)	Nonresponders (n = 88)	*P* Value
Biological sex	154 (75%)	64 (73%)	.618
Age at injury∗	56 (18–75)	56 (18–74)	.572
Injured dominant hand	87 (43%)	38 (43%)	.932
Operative treatment	80 (39%)	40 (45%)	.320
*Quick*DASH	4.5 (0–14)	4.5 (0–20)	.728
PCS	10 (4–18)		
GSE	30 (28–34)		
HADS-A	3 (1–6)		
HADS-D	1.2 (1–3.5)		

Values presented as n, % or median, IQR.

GSE, General Self-Efficacy Scale; HADS-A, Hospital Anxiety and Depression Scale (anxiety); HADS-D, Hospital Anxiety and Depression Scale (depression); IQR, interquartile range; PCS, Pain Catastrophizing Scale; *Quick*DASH, Disabilities of the Arm, Shoulder, and Hand.

∗ Age is presented as mean, range.

Thirty percent (n = 88) were nonresponders. The nonresponders did not differ from the study patients in our cohort (Table 1).

### Clinical outcome

In bivariate analysis, all measured psychological questionnaires correlated with the *Quick*DASH over a decade after the injury (Table 2). Correlations were weak to moderate. Patients with worse *Quick*DASH scores reported higher levels of pain catastrophizing, depression, and anxiety, as well as lower self-efficacy.

**Table 2:** Bivariate Correlations for Psychological Factors and *Quick*DASH

Variable	*Quick*DASH		
	Spearman’s Rho	Strength of Correlation	*P* Value
PCS	0.35	Moderate	<.001
GSE	−0.23	Weak	.001
HADS-A	0.22	Weak	.001
HADS-D	0.20	Weak	.003

GSE, General Self-Efficacy Scale; HADS-A, Hospital Anxiety and Depression Scale (anxiety); HADS-D, Hospital Anxiety and Depression Scale (depression); PCS, Pain Catastrophizing Scale; *Quick*DASH, Disabilities of the Arm, Shoulder, and Hand.

Among the factors included in the multivariable analysis, higher age (partial R^2^ = 0.04; *P* = .005), having injured the dominant hand (partial R^2^ = 0.02; *P* = .045), and greater pain catastrophizing (partial R^2^ = 0.13; *P* <.001) were associated with an increase in *Quick*DASH score at the 11–13-year follow-up (Table 3).

**Table 3:** Multivariable Analysis of Predictive Factors and Limitations at the 11- to 13-Year Follow-Up∗

Variables Affecting *Quick*DASH	Regression Coefficient	Standard Error	*P* Value	VIF	Partial R^2^
Age at injury	0.03 (0.01–0.05)	0.01	.005	1.06	0.04
Injured dominant hand	0.53 (0.01–1.05)	0.26	.045	1.00	0.02
PCS	0.08 (0.05–0.10)	0.01	<.001	1.05	0.13

The adjusted R^2^ for the model is 0.19. The values are given as the regression coefficient, with 95% CIs in parentheses. The table only displays variables with a significance of *P* < .05.

CI, confidence interval; *Quick*DASH, Disabilities of the Arm, Shoulder, and Hand; VIF, variable inflation factor.

∗ The model examined age, female sex, injured dominant hand, PCS, and operative treatment.

## Discussion

The objective of this study was to assess the significance of clinical and psychosocial factors assessed over a 13-year period postinjury on limitations measured using PROMs 11–13 years after a DRF. The findings enhance the understanding of psychological factors and their long-term effects on patients’ perceived outcomes. The primary factors contributing to longer term limitations at 11–13 years postinjury were increased pain catastrophizing, patient age, and injury to the dominant hand.

The linear regression model explained approximately 20% of the variance in the *Quick*DASH. The PCS was the single most important factor, alone accounting for 13% of the variance. A recent article on the same cohort exploring radiographic factors found no correlation between radiographic alignment and clinical outcome (range of motion, grip strength, and *Quick*DASH).^5^ Thus, psychological factors appear to be more important for the variance in *Quick*DASH than any radiological factors 11–13 years after injury.

This study adds to the existing body of research indicating that psychosocial factors play a major role in the variability of PROMs.^11^^,^^13^, ^14^, ^15^, ^16^, ^17^, ^18^, ^19^, ^20^ The assessment of coping strategies in response to pain (such as the PCS) is likely assessing an underlying dimension of human illness behavior that adversely affects functional limitations following upper limb conditions.^28^ Achieving optimal outcomes extends beyond merely reconstructing anatomy and concentrating on the technical facets of fracture management.^11^ Evidence suggests that skills training in optimizing recovery in orthopedic patients is feasible and may benefit recovery.^29^ However, measuring and attempting to optimize outcomes based on psychosocial factors may present challenges. The existing evidence shows that patients with poorer psychosocial profiles experience worse PROMs at baseline.^30^ Measuring and using psychosocial factors to optimize outcome may however prove problematic. Furthermore, despite receiving the same level of benefit from treatment as those with more favorable psychosocial profiles, their ‘return to baseline’ indicates that they continue to score poorly based on PROMs.^30^^,^^31^ Consequently, should skills training demonstrate efficacy, enhancing outcomes after a DRF through such training or similar coping strategies is likely to modify the patients’ baseline rather than amplify the benefits of their orthopedic treatment. If this is the case, all patients with poorer psychosocial profiles (regardless of whether they have sustained a DRF) would benefit from skills training, making it less relevant to orthopedic treatments and discussions.

The study has several limitations. The main limitation is the *Quick*DASH, which, although frequently used and validated for DRFs, is not a wrist-specific questionnaire.^24^ Moreover, *Quick*DASH suffers from floor effects.^32^ Because of this and given that *Quick*DASH seem to be primarily measuring psychosocial factors, there might be a need for better outcome measures after DRFs.^11^ According to the treatment algorithm used in this study, fractures that remained displaced after reduction or were displaced at follow-up were offered surgical treatment. Consequently, most fractures in this cohort exhibited relatively good alignment. Thus, the occurrence of severe malunions in this cohort is limited, and the generalizability of these findings may not extend to fractures that heal in a severely malunited position.^33^ The observational study design does not enable us to determine the superiority of any treatment method. Regrettably, we lost 30% to follow-up; however, the nonresponders did not differ from our cohort, suggesting that they did not affect the results. In addition, the variables in the present study only explained a limited amount of variance in the *Quick*DASH scores. As such, there are other unknown variables that also influence outcomes. In addition, this study fails to provide information on the change of psychological factors over time and, notably, the absence of baseline values before injury for all questionnaires. Although most patients completed the PROMs in person, some assessments were conducted over the phone. Moreover, the PCS, GSE, HADS-A, and HADS-D were collected approximately 1 year after the *Quick*DASH. These limitations may have introduced procedural, measurement, and responder bias.

In conclusion, a decade after sustaining a DRF, patients with higher scores on the PCS reported poorer outcomes as measured by the *Quick*DASH. The PCS accounts for 13% of the observed variability in the *Quick*DASH. This finding suggests that psychological factors impact PROMs more than radiological factors in the long-term.

## Conflicts of Interest

No benefits in any form have been received or will be received related directly to this article.
